# Abnormal interlimb coordination of motor developmental delay during infant crawling based on kinematic synergy analysis

**DOI:** 10.1186/s12938-024-01207-1

**Published:** 2024-02-07

**Authors:** Li Zhang, Chong Xu, Lin Chen, Yuan Liu, Nong Xiao, Xiaoying Wu, Yuxia Chen, Wensheng Hou

**Affiliations:** 1 Key Laboratory of Biorheological Science and Technology, Ministry of Education, Bioengineering College, Chongqing University, Chongqing, 400044 China; 2 Chongqing Engineering Research Center of Medical Electronics Technology, Chongqing, 400044 China; 3 Department of Rehabilitation Center, Children’s Hospital, Chongqing Medical University, Chongqing, 400014 China

**Keywords:** Kinematic synergies, Interlimb coordination, Motor developmental delay, Infant crawling, Non-negative matrix factorization

## Abstract

**Background:**

Previous studies have reported that abnormal interlimb coordination is a typical characteristic of motor developmental delay (MDD) during human movement, which can be visually manifested as abnormal motor postures. Clinically, the scale assessments are usually used to evaluate interlimb coordination, but they rely heavily on the subjective judgements of therapists and lack quantitative analysis. In addition, although abnormal interlimb coordination of MDD have been studied, it is still unclear how this abnormality is manifested in physiology-related kinematic features.

**Objectives:**

This study aimed to evaluate how abnormal interlimb coordination of MDD during infant crawling was manifested in the stability of joints and limbs, activation levels of synergies and intrasubject consistency from the kinematic synergies of tangential velocities of joints perspective.

**Methods:**

Tangential velocities of bilateral shoulder, elbow, wrist, hip, knee and ankle over time were computed from recorded three-dimensional joint trajectories in 40 infants with MDD [16 infants at risk of developmental delay, 11 infants at high risk of developmental delay, 13 infants with confirmed developmental delay (CDD group)] and 20 typically developing infants during hands-and-knees crawling. Kinematic synergies and corresponding activation coefficients were derived from those joint velocities using the non-negative matrix factorization algorithm. The variability accounted for yielded by those synergies and activation coefficients, and the synergy weightings in those synergies were used to measure the stability of joints and limbs. To quantify the activation levels of those synergies, the full width at half maximum and center of activity of activation coefficients were calculated. In addition, the intrasubject consistency was measured by the cosine similarity of those synergies and activation coefficients.

**Results:**

Interlimb coordination patterns during infant crawling were the combinations of four types of single-limb movements, which represent the dominance of each of the four limbs. MDD mainly reduced the stability of joints and limbs, and induced the abnormal activation levels of those synergies. Meanwhile, MDD generally reduced the intrasubject consistency, especially in CDD group.

**Conclusions:**

These features have the potential for quantitatively evaluating abnormal interlimb coordination in assisting the clinical diagnosis and motor rehabilitation of MDD.

## Introduction

Organized and rhythmic interlimb coordination during hands-and-knees crawling is usually regarded as a sign of typical development of motor function for infants [1]. However, for infants with motor developmental delay (MDD), crawling can be challenging and even impossible to accomplish due to abnormal interlimb coordination [2]. MDD is a special developmental disorder, which is generally caused by prematurity, low birth weight, neonatal seizures, or/and other risk factors [3, 4]. MDD may further progress to global developmental delay (GDD), cerebral palsy (CP) or a specific neuromuscular disorder with the increase in biological ages for infants/children [4]. Nevertheless, the extent to which MDD is related to delayed/impaired motor control of the central nervous system (CNS) cannot be ascertained [2, 3]. Joint velocities regulated by the CNS have been widely used to evaluate interlimb coordination and motor function, and kinematic synergy among multiple joints of limbs has been proposed as a control strategy for interlimb coordination and motor function [5–7]. Furthermore, strong stability of joints and limbs [8], appropriate activation levels of synergies [9] and high intrasubject consistency [10] appear to be the typical kinematic features to describe well interlimb coordination during human movement. Although the impacts of MDD on interlimb coordination have been observed among the joint velocities of limbs during infant crawling [2, 11], how abnormal interlimb coordination of MDD during infant crawling is manifested in the kinematic features (e.g., the stability of joints and limbs, activation levels of synergies and intrasubject consistency) based on kinematic synergy analysis is still an open question.

Accurate assessment of abnormal interlimb coordination during crawling is beneficial for clinicians to establish a set of personalized rehabilitation schedules for MDD in infant’s early life [4]. In clinical practice, clinicians usually use the parents’ reports and their own observations to detect a possible MDD of infants/children [3, 4]. The scale assessments, such as Bayley Scales of Infant Development II (BSID-II), Peabody Developmental Motor Scales, 2nd edition (PDMS-2), Toddler Infant Motor Evaluation (T.I.M.E.), Pediatric Evaluation of Disability Inventory (PEDI) and Gross Motor Function Measure (GMFM-88/GMFM-66), are commonly used to measure the development of motor function, as well as interlimb coordination of infants and children [12]. Combined with these assessments, MDD can be clinically recognized and rated, but they are normally based on the subjective and observational analysis of abilities as infants/children perform numerous tasks [12, 13]. Apart from these, the time- and frequency-domain parameters of surface electromyography (sEMG) signals recorded from muscle groups, such as muscular co-activation index and muscle synergy, are considered effective for the analysis of crawling movement [14–16]. However, sEMG-based parameters cannot directly reflect the coordination of joints and limbs, and may be challenging the requirement of robustness for complicated limb movement [17]. In addition, kinematic parameters, such as velocity, cadence, duration, angle and smoothness, can provide important information regarding the quality of crawling movement [2, 18, 19]. In spite of various kinematic parameters evaluating interlimb coordination, joint velocity is the crucial factor affecting interlimb coordination during crawling, and the changes in other kinematic parameters are related to the changing joint velocities over time [1, 6, 19]. Currently, studies of the effects of joint velocities on interlimb coordination during infant crawling are still scarce [5], as are studies analyzing abnormal interlimb coordination in MDD due to delayed/impaired motor control of the CNS.

Kinematic synergy analysis is regarded as a valid method to explore the underlying motor control strategies by which the CNS regulates interlimb coordination of limb movement [7]. There is abundant evidence supporting the view that the CNS exploits a limited number of modules combined by multiple motor units (MUs), called “synergies”, to simplify the production of limb movement [7, 20]. Computational techniques, especially matrix factorization algorithms, have been widely used to derive synergies to further investigate different motor control strategies of the CNS [21–23]. Non-negative matrix factorization (NMF) is the most commonly used algorithm for synergy extraction due to the assumption of non-negativity for all matrices (i.e., original and decomposed matrices) [23–25]. NMF can decompose the multi-joint velocities for limb movement into two components: the time-invariant kinematic synergies combining by all joints with different weightings, and the time-varying activation coefficients representing the activation patterns of corresponding synergies [22, 26]. Kato et al. successfully employed the NMF algorithm to derive kinematic synergies from the tangential velocities of joints of the four limbs for spontaneous movements of infants, where these synergies represented the dominance of different combinations of limbs [27]. Since the tangential velocities of joints do not include the direction of limb movement in space, the analytical complexity of limb movement can be highly reduced [27, 28]. Although our pilot study has preliminarily distinguished infants with MDD from typically developing (TD) infants by the kinematic synergies of tangential velocities of joints during crawling [29], it is less clear how MDD affects interlimb coordination based on those synergies.

Under the synergy analysis framework, researchers have proposed a few kinematic features to objectively quantify interlimb coordination during human movement. For instance, the variability accounted for (VAF) as calculated from original and decomposed matrices and the synergy weightings of each MU in the derived synergies have been used to measure the stability of joints and limbs for limb movement, which can be changed in individuals with neurological diseases [8, 27]. During human movement, the full width at half maximum (FWHM) and center of activity (CoA) of activation coefficients can effectively characterize the differences in the activation levels of corresponding synergies not only between TD children and children with neurological diseases but also among various motor behaviors [9, 30, 31]. Moreover, the cosine similarity (CS) of synergies and activation coefficients for limb movement has been shown to be effective for evaluating the intrasubject consistency of healthy individuals, who exhibit high repeatability in the motor control strategy of the CNS [10, 32]. Nevertheless, to our knowledge, the above kinematic features, which are crucial measures of joint activities for clinical diagnosis and motor rehabilitation of MDD, are used only to quantify interlimb coordination of muscular activities during human movement. Our previous study demonstrated that the kinematic features calculated from kinematic synergies during crawling could reflect the abnormal organization of joint activities for infants with MDD [33], but it quantified only abnormal coordination among the joints of each of the four limbs, providing little information about abnormal interlimb coordination across the joints of the four limbs during crawling.

The aim of the current study was to evaluate how abnormal interlimb coordination of MDD during infant crawling was manifested in the stability of joints and limbs, activation levels of synergies and intrasubject consistency from the aspect of kinematic synergies of tangential velocities of joints. Given the modular control hypothesis of kinematic synergy, the global recruitment pattern among the four limbs should be combined by multiple synchronous recruitment patterns of a specific set of limbs [27, 34]. As a result, we first assumed that interlimb coordination patterns of joints and limbs during infant crawling could be represented with the tangential velocities of joints, and these synergies would be dominated by different combinations of limbs. Then, based on these synergies, we assumed that abnormal interlimb coordination of MDD could be reflected in the decrease in the stability of joints and limbs, abnormal activation levels of synergies and the decrease in the intrasubject consistency, and these manifestations would be related to impaired/immature motor function and selective motor control.

## Results

Although according to GMFM-88, the scaled scores of crawling/kneeling dimension were similar ($\chi^{2} \left( 3 \right) = {5}{\text{.436}}, p{ = 0}{\text{.143}}$) among TD infants [TD group: median scores 54(3.5)], infants at risk of developmental delay [ARDD group: median scores 51(7.75)], infants at high risk of developmental delay [AHRDD group: median scores 54(6)] and infants with confirmed developmental delay [CDD group: median scores 54(10)], the development of motor function for infants with MDD was indeed slower than that for TD infants at the same ages. Since the changes of MDD from infancy to adulthood were progressive, we compared the relative rather than absolute differences among those four groups to evaluate abnormal interlimb coordination of MDD during infant crawling.

### Four kinematic synergies extracted from tangential velocities of joints

**Fig. 1:**
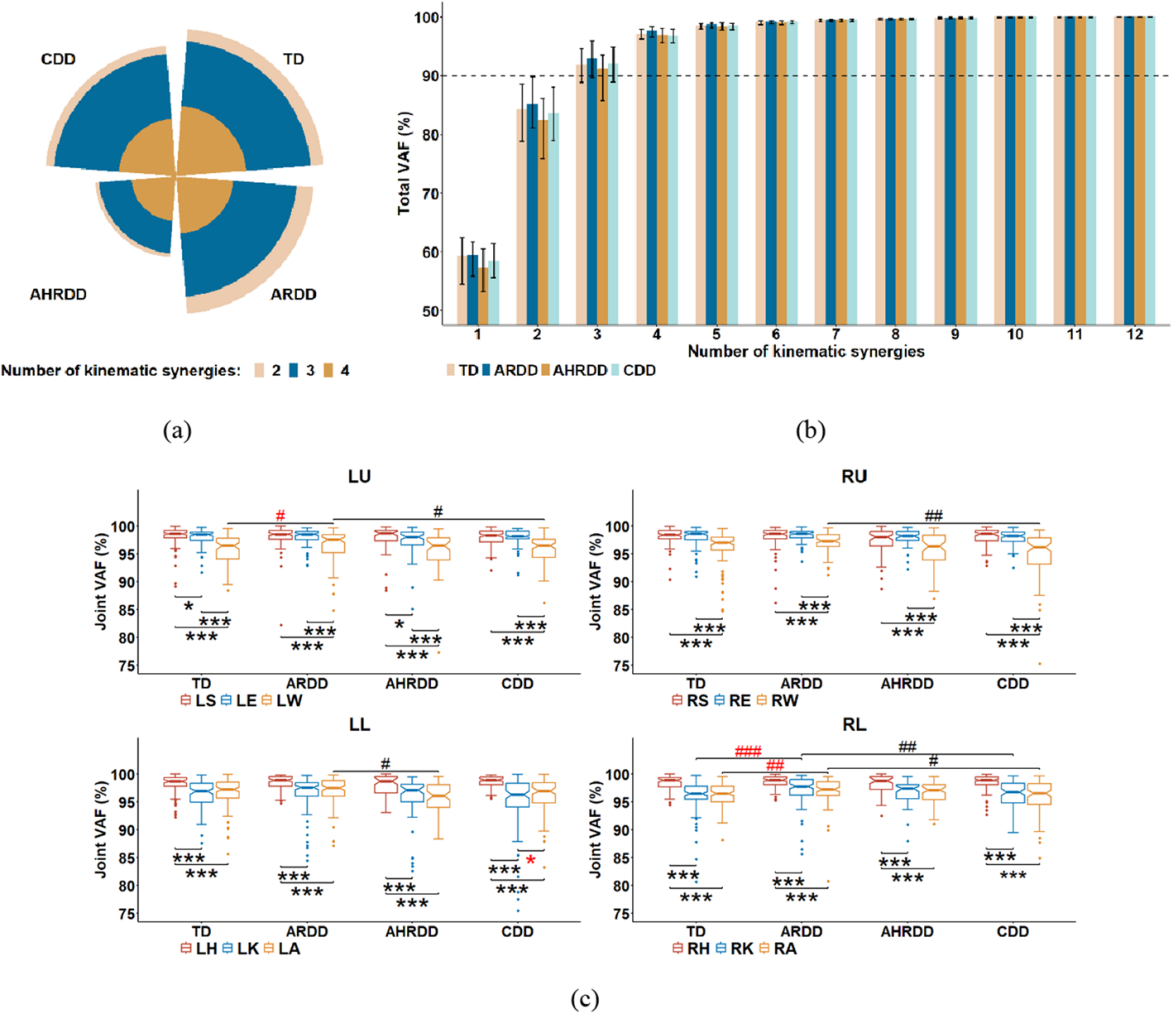
**a** Identified number of kinematic synergies, **b** percentages of total VAF (median, 25th and 75th percentiles) with the increasing number of kinematic synergies from 1 to 12, and (**c**) percentages of joint VAF yielded by the four kinematic synergies for the four groups. The dotted line in (**b**) shows 90% threshold. Black *S > E > W or H > K > A; otherwise, red *(Wilcoxon signed-rank test). Black #TD > ARDD > AHRDD > CDD; otherwise, red # [Kruskal–Wallis test (Tukey post hoc)]. */#*p* < 0.05, **/##*p* < 0.01, ***/###*p* < 0.001. L/RU: left/right upper limbs, L/RL: left/right lower limbs, S: shoulder, E: elbow, W: wrist, H: hip, K: knee, A: ankle

According to the selection criteria for synergy number (total VAF > 90% and joint VAF > 75% and the increase in mean values of joint VAF for all joints < 5%) defined by a previous study [8], all infants for the four groups identified 2–4 kinematic synergies, as shown in Fig. 1 and Table 1. In addition, no significant difference of the identified number of kinematic synergies among those four groups ($\chi^{2} \left( 3 \right) = {3}{\text{.539, }}p\; = \;0.316$) was detected by Kruskal–Wallis one-way ANOVA. Therefore, the number of kinematic synergies for the four groups was set to four for subsequent analyses.

**Table 1:** Increase in mean values of joint VAF for all joints for the four groups

Group	∆1,2	∆2,3	∆3,4	∆4,5	∆5,6	∆6,7	∆7,8	∆8,9	∆9,10	∆10,11	∆11,12
TD	31.094 (6.226)	7.297 (4.377)	4.311 (4.104)	1.177 (0.804)	0.583 (0.375)	0.322 (0.250)	0.191 (0.122)	0.124 (0.107)	0.080 (0.082)	0.042 (0.053)	0.030 (0.032)
ARDD	34.336 (6.077)	7.116 (3.773)	3.967 (3.407)	0.892 (1.025)	0.453 (0.337)	0.311 (0.226)	0.185 (0.159)	0.123 (0.107)	0.075 (0.068)	0.043 (0.051)	0.033 (0.024)
AHRDD	32.004 (6.043)	7.894 (4.614)	4.703 (3.754)	1.347 (1.058)	0.667 (0.688)	0.381 (0.204)	0.188 (0.179)	0.129 (0.113)	0.096 (0.100)	0.047 (0.061)	0.039 (0.038)
CDD	32.188 (7.517)	7.545 (5.145)	4.058 (3.645)	1.501 (1.277)	0.607 (0.509)	0.315 (0.270)	0.197 (0.128)	0.113 (0.073)	0.069 (0.065)	0.033 (0.039)	0.029 (0.032)

Δ*i*, (*i* + 1): number of kinematic synergies increasing from *i* to (*i* + 1).* i* ranges from 1 to 11. Data are expressed as median (interquartile range). Interquartile range = 75th percentile ‒ 25th percentile

Furthermore, as shown in Fig. 1c, the proximal joints in upper (i.e., shoulder and elbow) or lower (i.e., hip) limbs for the four groups showed significantly higher joint VAF values than the distal joints (i.e., wrist or knee and ankle), even if there were significant differences between LS and LE in TD and AHRDD groups or between LK and LA in CDD group (all *p* < 0.05). These results indicated that the changes in the tangential velocity profiles of proximal joints during infant crawling could be captured more effectively than those of distal joints. At the same time, ARDD group exhibited significantly higher joint VAF values than: (1) TD group in the LW, RK and RA; (2) AHRDD group in the LA; (3) CDD group in the L/RW, RK and RA (all *p* < 0.05). That is, MDD would cause insufficient organization of distal joints during infant crawling.

### Comparison of synergy weightings in the four extracted kinematic synergies

**Fig. 2:**
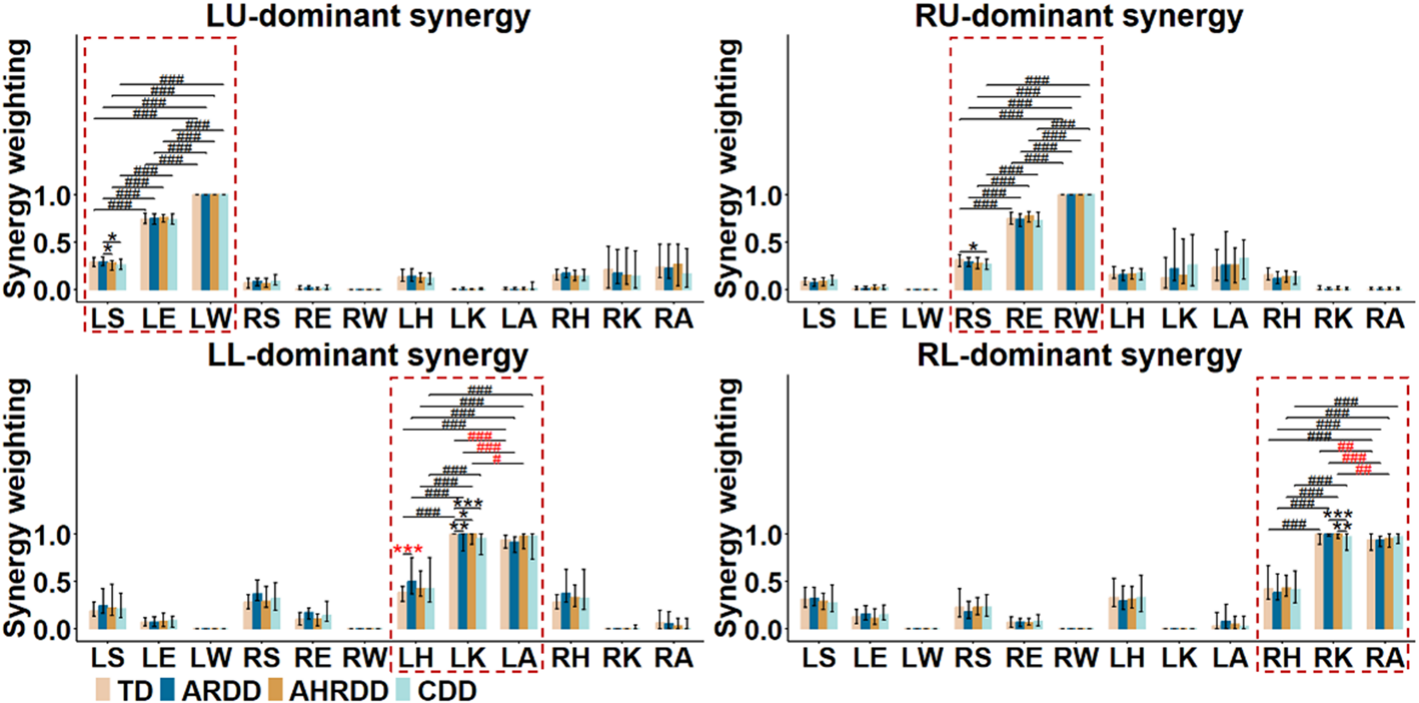
Synergy weightings (median, 25th and 75th percentiles) in the four extracted kinematic synergies for the four groups. Black *TD > ARDD > AHRDD > CDD; otherwise, red *[Kruskal–Wallis test (Tukey post hoc)]. Black #S < E < W or H < K < A; otherwise, red # (paired *t* test or Wilcoxon signed-rank test). */#*p* < 0.05, **/##*p* < 0.01, ***/###*p* < 0.001

Figure 2 shows the synergy weightings in the four extracted kinematic synergies for the four groups. Although a fixed-order kinematic synergy for any infant was not bound to be dominated by a particular limb, these synergies represented the dominance of each of the four limbs were referred to as the limb-dominant synergies (i.e., LU-, RU-, LL- and RL-dominant synergies). Moreover, despite significant differences of synergy weightings between the shoulder and elbow for the four groups or between the knee and ankle in TD, ARDD and AHRDD groups, the distal joints (i.e., wrist or knee and ankle) for the four groups showed significantly higher synergy weightings than the proximal joints (i.e., shoulder and elbow or hip) (all *p* < 0.05), indicating larger contribution of distal joints to the crawling movement.

In addition, the synergy weightings in TD group were significantly higher than those of RS in CDD group and of LK in ARDD, AHRDD and CDD groups, but were significantly lower than those of LH in ARDD group (all *p* < 0.05). The synergy weightings in ARDD group were significantly higher than those of LS in AHRDD and CDD groups, and of RK in CDD group (all *p* < 0.05). AHRDD group exhibited significantly higher synergy weightings of RK than CDD group (*p* = 0.008). These results indicated that MDD mainly reduced the contribution of shoulder and knee to the crawling movement.

### Comparison of activation levels of the four extracted kinematic synergies

**Fig. 3:**
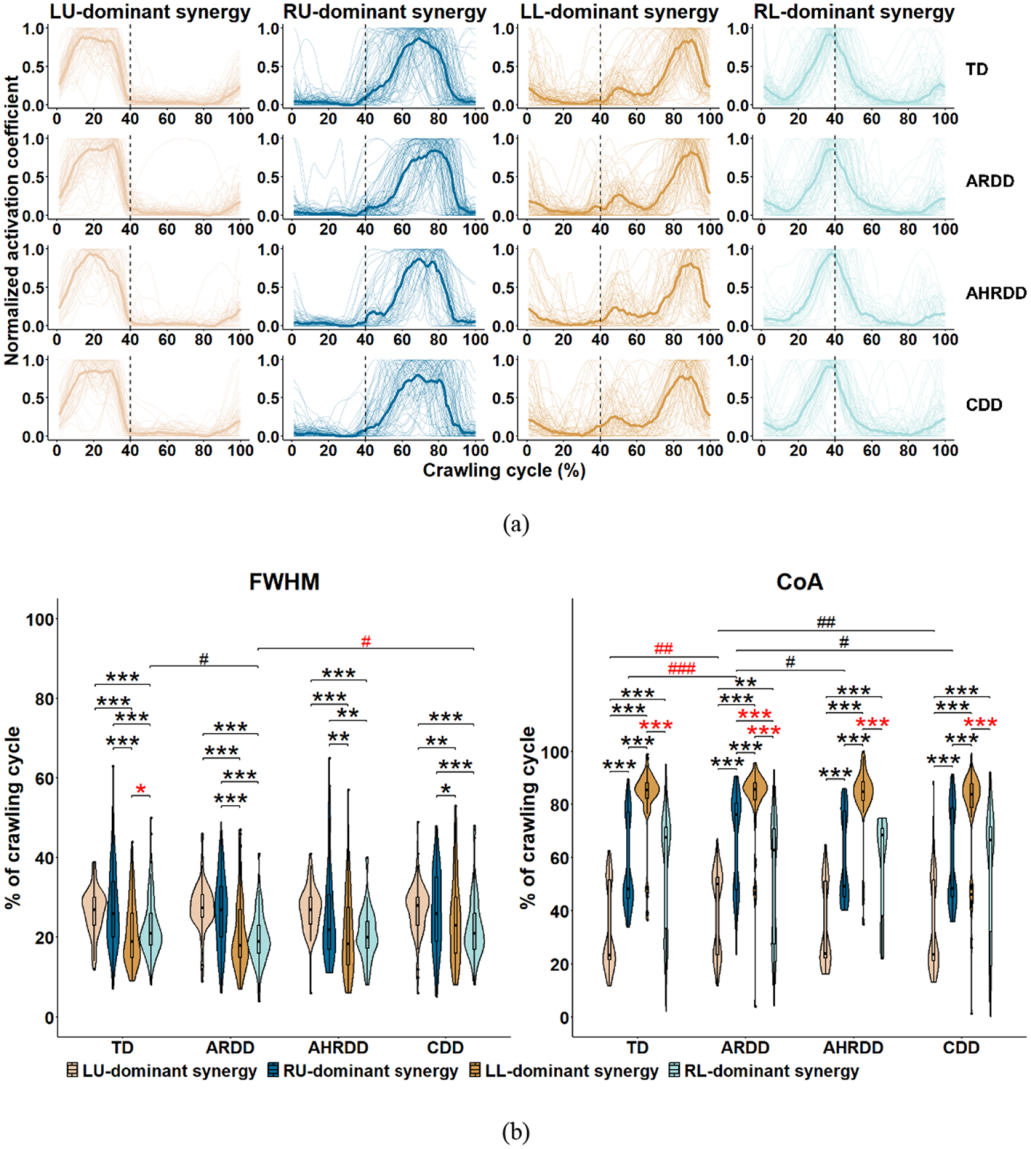
**a** Normalized activation coefficient curves of the four extracted kinematic synergies, and their (**b**) FWHM and CoA values for the four groups. Each thick solid line denotes the median of activation coefficient curves corresponding to the kinematic synergy in each group. Vertical dotted lines separate the swing and stance phases during a crawling cycle. For FWHM, black *LU-dominant synergy > RU-dominant synergy > LL-dominant synergy > RL-dominant synergy; otherwise, red *(paired *t* test or Wilcoxon signed-rank test). The meanings of black/red *for CoA are opposite to those for FWHM. Black #TD > ARDD > AHRDD > CDD; otherwise, red # [Kruskal–Wallis test (Tukey post hoc)]. */#*p* < 0.05, **/##*p* < 0.01, ***/###*p* < 0.001

To ascertain the trends of variation in the activation coefficient curves, every curve during a crawling cycle was normalized to its maximum value, as shown in Fig. 3a. By visual inspection, the activation of limb-dominant synergies for the four groups during a crawling cycle occurred in a roughly consistent sequence, with LU-, RL-, RU- and LL-dominant synergies appearing successively.

Figure 3b shows the FWHM and CoA values of activation coefficient curves for the four groups. Despite the significantly difference of FWHM values between the LL- and RL-dominant synergies in TD group, the LU- and RU-dominant synergies showed significantly higher FWHM values than the LL- and RL-dominant synergies for the four groups (all *p* < 0.05), indicating strong participation of upper limbs in the crawling movement. With regard to the CoA values, the broadly gradual increase in LU-, RL-, RU- and LL-dominant synergies for the four groups (all *p* < 0.01) agreed with Fig. 3a, even if no significant differences between the RU- and RL-dominant synergies were found in TD, AHRDD and CDD groups. In addition, ARDD group showed: (1) significantly lower FWHM values of RL-dominant synergy than TD and CDD group; (2) significantly higher CoA values of LU-dominant synergy than TD and CDD groups, and of RU-dominant synergy than TD, AHRDD and CDD groups (all *p* < 0.05). These results indicated that MDD mainly widened the activation of RL-dominant synergy and delayed the activation of LU- and RU-dominant synergies.

### Comparison of intrasubject consistency based on the four extracted kinematic synergies

**Fig. 4:**
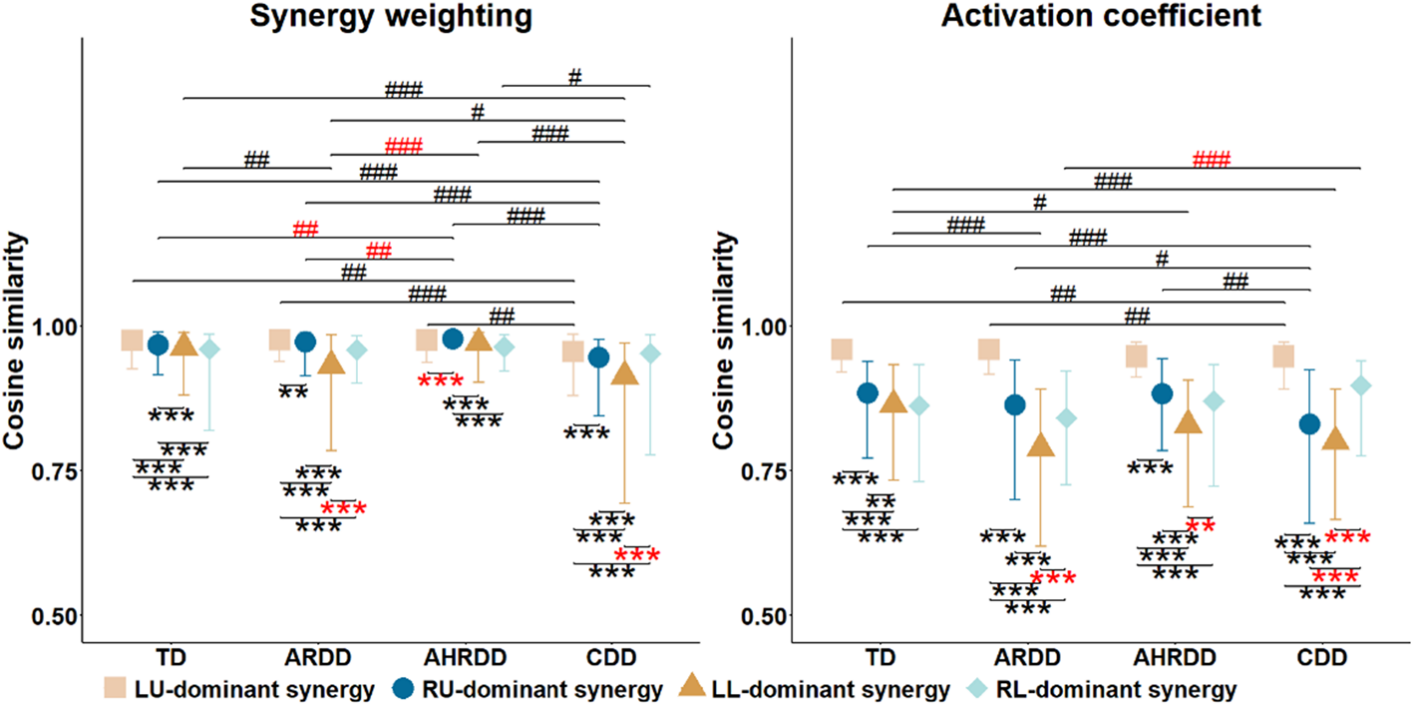
Cosine similarity (median, 25th and 75th percentiles) of synergy weightings and activation coefficients for the four groups. Black *LU-dominant synergy > RU-dominant synergy > LL-dominant synergy > RL-dominant synergy; otherwise, red *(Wilcoxon signed-rank test). Black #TD > ARDD > AHRDD > CDD; otherwise, red #[Kruskal–Wallis test (Tukey post hoc)]. */#*p* < 0.05, **/##*p* < 0.01, ***/###*p* < 0.001

Figure 4 illustrates the CS values separately calculated from synergy weightings and activation coefficients for the four groups. For either synergy weightings or activation coefficients, although the CS values of LU- and RU-dominant synergies were not always significantly higher than those of LL- and RL-dominant synergies for the four groups, these comparative relationships, by and large, could be observed, indicating better intrasubject consistency of upper limbs. At the same time, it is possible to observe a significant difference of CS values of synergy weightings or activation coefficients between the LU- and RU-dominant synergies or between the LL- and RL-dominant synergies in TD, ARDD, AHRDD or CDD group, suggesting inconsistent coordination patterns of bilateral limbs.

In addition, compared to TD, ARDD and AHRDD groups, CDD group showed a decreasing trend of CS values of synergy weightings and activation coefficients, even though these comparative relationships were not always significant or were reversed. These results demonstrated that CDD group exhibited markedly poor intrasubject consistency during infant crawling. Meanwhile, the significant differences of CS values of synergy weightings or activation coefficients among TD, ARDD and AHRDD groups were also observed in RU- or LL-dominant synergies, suggesting the immature coordination development of contralateral RU and LL.

## Discussion

The primary objective of this study was to explore how abnormal interlimb coordination of MDD during infant crawling was manifested in the stability of joints and limbs, activation levels of synergies and intrasubject consistency based on kinematic synergies of tangential velocities of joints. The present work showed that: (1) four kinematic synergies dominated by each of the four limbs sufficiently represented the dynamic profiles of tangential velocities of joints; (2) compared to distal joints, the proximal joints captured the changes in the tangential velocity profiles more effectively and exhibited smaller contribution to the crawling movement; (3) MDD mainly caused insufficient organization of distal joints, reduced the contribution of shoulder and knee to the crawling movement, as well as widened the activation of RL-dominant synergy and delayed the activation LU- and RU-dominant synergies; meanwhile, the markedly poor intrasubject consistency were also found in CDD group. The above results were further explained in the following subsections.

### Synergistic combination of single-limb movements for crawling movement

During infants crawling on hands and knees, four kinematic synergies were extracted from the tangential velocities of joints, and these synergies were dominated by each of the four limbs (Figs. 1, 2, Table 1). The dominance of these four extracted kinematic synergies was consistent with the results during infants’ spontaneous movements [27], representing four types of single-limb movements. However, these results in [27] were obtained from the wrist and knee of limbs, and took TD infants as objects. Extending to the current study, these results were not only validated in the major joints (i.e., shoulder, elbow, wrist, hip, knee and ankle) of limbs for TD infants during crawling, but also demonstrated in infants with different degrees of MDD (i.e., ARDD, AHRDD and CDD groups). Thereupon, we wonder if the crawling movement might be generated by the interaction of multiple joints independently controlling each of the four limbs.

In fact, crawling requires multiple joints and limbs to cooperatively participate in motor control to move body forward [11]. As shown in Fig. 3, the LU-, RL-, RU- and LL-dominant synergies for the four groups were activated in sequence from the swing to stance phase, and the CoA values of LU-, RL-, RU- and LL-dominant synergies gradually increased. These results were in line with previous findings that most infants tended to use trot-like coordination patterns (i.e., moved diagonal limbs together and alternated ipsilateral limbs) during crawling [1, 5]. During crawling, specialized neural circuits organize interlimb coordination patterns, which may be provided via the mechanical coupling of arm-leg movements in terms of segmental motor control of the CNS rather than in terms of individual limb control [15]. Moreover, it was obvious that these four kinematic synergies representing the dominant movements of each of the four limbs were not restricted to the orders extracted by the NMF algorithm (Fig. 2), which indicated a variety of synergistic patterns with different combinations of interlimb coordination based on a common principle underlying neural control. According to synergy theory, kinematic synergies represent a library of motion subtasks, which can be combined synergistically and flexibly by the CNS to produce complex and purposeful movements [7, 20]. Combined with our results, it was suggested that interlimb coordination patterns during infant crawling were not composed of multiple synergistic patterns independently controlled by each of the four limbs, but combined a variety of single-limb movements that were synchronously controlled by the CNS. As a consequence, in addition to provide novel insight into interlimb coordination, the kinematic synergies of tangential velocities of joints had the potential to reveal the CNS control strategy underlying abnormal interlimb coordination of MDD during infant crawling.

### Reduced stability of joints and limbs for infants with MDD

Compared to distal joints, the proximal joints captured the changes in the tangential velocity profiles during infant crawling more effectively (i.e., higher joint VAF values of proximal joints) (Fig. 1c), validating the principle that motor function developed from the proximal to distal joints for human body [34]. Moreover, the results obtained from synergy weighting in current study (Fig. 2) agreed with our pilot study that extracted the kinematic synergies from the joint velocities of each of the four limbs during infant crawling [29], which confirmed that the distal joints exhibited larger contribution to the crawling movement (i.e., higher synergy weightings of distal joints) than the proximal joints. Thus, the different results of joint VAF values and synergy weighting between proximal and distal joints were not contradictory, but were complementary and represented different physiological meanings. In addition, this discrepancy could be due to the different functions of proximal and distal joints for limb movement [35]. That is, compared to proximal joints, the distal joints had relatively later development of motor function, but played crucial roles in the completion of crawling movement. In addition, the significant difference of joint VAF values or synergy weightings between the proximal joints of upper limbs or between the distal joints of lower limbs in TD, ARDD, AHRDD or CDD group (Figs. 1c, 2) could be the result of different functional control mechanisms of joints and limbs related to the immature development of motor function for infants [6, 14]. Therefore, the different functions of proximal and distal joints for infants could reflect the stability of joints and limbs controlled by the CNS to produce a coordinated crawling movement [6, 11].

However, infants with MDD, who may be further diagnosed with nervous system diseases, such as GDD and CP, had insufficient organization of distal joints (i.e., reduced joint VAF values of distal joints) as well as the reduced contribution of shoulder and knee to the crawling movement (i.e., reduced synergy weightings of shoulder and knee) (Figs. 1c, 2). These results could be attributed to the delayed/impaired motor control of the CNS for MDD, which would result in abnormal descending motor commands and thus affected the stability of joints and limbs during crawling [2, 3]. Specifically, since the limb movement can be approximated as the rotation of a rigid body about a fixed point, the development of motor function for distal joints is more susceptible to the state of the nervous system than that of proximal joints [34–37]. In addition, researchers have pointed out that the reduced contribution of shoulder may be a matter of strategy choice; that is, children who were unable to accurately accomplish the motor behaviors in their distal joints might compensate using motor function of proximal joints to produce a smooth and rhythmical motion [35]. Nevertheless, it was worth noting that the comparative relationships of joint VAF values and synergy weightings were not always significant among those four groups and even reversed between TD and ARDD groups (Figs. 1c, 2). Thereinto, those no significant differences could be interpreted as the natural functional redistribution among multiple joints of limbs to propel and control a given motion task [38]. Those opposite differences between TD and ARDD groups were related to the improvement in motion proficiency for ARDD group after rehabilitation training, even though their motor abilities should be similar because of similar biological ages and delayed ages of gross motor [2, 33]. Taken together, these reductions in the joint VAF values and synergy weightings for infants with MDD reflected the reduced stability of joints and limbs during crawling, which could be used in the future for the development of rehabilitation strategies.

### Abnormal activation levels of limb-dominant synergies for infants with MDD

The upper limbs showed stronger participation and better intrasubject consistency (i.e., higher FWHM and CS values of LU- and RU-dominant synergies) during infant crawling than the lower limbs (Figs. 3b, 4), validating the asynchronous developmental sequence of upper to lower limbs [1, 11, 33]. Moreover, our study further found that MDD widened the activation duration of RL-dominant synergy (i.e., increased FWHM values of RL-dominant synergy) during infant crawling (Fig. 3b). These results were in line with our previous study based on kinematic synergy analysis among the joints of each of the four limbs during crawling, implying that the coordination patterns of lower limbs were much more affected by the motor developmental levels for infants [33]. This influence might be related to the nervous system’s need for additional efforts to maintain functionality and cope with continuous perturbations [30, 31]. At the same time, more pronounced effects of MDD on the RL-dominant synergy rather than the LL-dominant synergy were also in agreement with previous studies investigating in healthy adults during walking, which could be possibly due to the principle of habitual leg, which would result in the differences in the muscle strength, stability, balance and proprioception of bilateral legs during human movement [39, 40]. In addition, such widened activation was compatible with prior findings regarding the muscle synergy analysis for children with CP and TD toddlers during walking, suggesting the immaturity of limb locomotor output [9]. Previous studies also reported that the broadened activation duration of synergies during human movement likely implied the increased metabolic cost and might limit the coordination of joints and limbs that required appropriate activation duration so as to adapt to different environments [25, 41]. Therefore, the potential mechanism for widened activation of RL-dominant synergy was related in part to delayed/impaired motor control of the CNS caused by MDD.

With regard to the CoA values of activation coefficients, the delayed activation of LU- and RU-dominant synergies in infants with MDD during crawling appeared to be the compensation mechanism of motor function, which could maintain the stable limb movement by blurring temporal boundaries to reduce the optimality and performance of joints and limbs [31, 42]. These results also agreed with a previous finding, in which Cappellini et al. observed that the CoA values increased with the increasing biological ages in TD children and decreased in children with CP, suggesting that the delayed activation of synergies was the result of brain immaturity or brain injury [9]. In fact, natural neuromuscular maturation, motor learning, rehabilitation treatment and environmental enrichment play important roles in the development of motor function [21, 43]. The differences between TD and ARDD groups or no difference among those four groups were also observed in the FWHM and CoA values of limb-dominant synergies (Fig. 3b), which could be partially attributed to the above factors affecting the maturity of motor skills. Whatever the exact mechanism of widened or delayed activation of limb-dominant synergies during infant crawling, they represented the characteristic features of abnormal activation levels of limb locomotor output in infants with MDD, which provided new evidence showing that the lack of maturation of limb locomotor output resulted in abnormal interlimb coordination.

### Reduced intrasubject consistency for infants with MDD during crawling

The coordination patterns of bilateral limbs in TD, ARDD, AHRDD and CDD groups were not quite consistent (i.e., inconsistent CS values between the LU- and RU-dominant synergies or between the LL- and RL-dominant synergies) (Fig. 4), which were contrary to the symmetry results of bilateral limbs of our previous study [33]. This discrepancy could be due to the differences of research perspectives. For the coordination patterns of bilateral limbs during infant crawling, our previous study measured the consistency based on the joints of each of the four limbs, while the current study was conducted across the joints of upper and lower limbs. In addition, these inconsistent coordination patterns of bilateral limbs in the current study might reflect varying postural control demands during a crawling cycle, depending on whether the left or right limb was leading to produce a smooth and rhythmical crawling movement [44]. At the same time, these inconsistent coordination patterns of bilateral limbs also implied the immature motor control of the CNS in infants’ early developmental stages, and the organization variability of joints and limbs was constrained by abnormal neuromuscular control strategy affected by MDD [2, 14]. Thereby, we speculated that MDD would impact the intrasubject consistency of bilateral limbs during infant crawling, but the influence likely varies.

Furthermore, by further comparing the intrasubject consistency during infant crawling among those four groups, we found that CDD group exhibited markedly poor intrasubject consistency (i.e., reduced CS values) (Fig. 4). According to the synergy theory, it is assumed that the CNS organizes interlimb coordination patterns, so that the motor control strategy improves the intrasubject consistency to achieve an accurate and stable limb movement [41]. Based on this hypothesis, the intrasubject consistency during crawling should increase with the increasing biological ages of infants. However, CDD group, due to relatively severe MDD and even have been diagnosed with CP, would result in the impaired execution of neural commands, and thus generated the reduced intrasubject consistency during infant crawling. This reduced intrasubject consistency were also in agreement with individuals with CP [9] and Parkinson [44] during walking, indicating the impaired motor control of the CNS. On the other hand, the reduced stability of joints and limbs and the abnormal activation levels of limb-dominant synergies for infants with MDD (Figs. 1c, 2, 3b) could also emerge as a result of reduced intrasubject consistency, suggesting some adjustments in motor details to maintain the repeatability and stability of crawling movement [32, 44]. In addition, the immature coordination development of contralateral RU and LL (i.e., significant differences of CS values of RU- or LL-dominant synergy) were also observed among TD, ARDD and AHRDD groups, which could be related to the immature development of motor function and the asymmetrical motor dysfunction caused by the damage to the brain nerves and cells [40]. In addition, the reason why this occurred in the contralateral RU and LL might be the segment of crawling cycles with the LW as the object, which would lead to the obvious coordination of contralateral LU and RL. Moreover, the CS values that were not always reduced in CDD group were relevant to the improvement in motion proficiency with the increasing biological ages for infants [11]. Taken together, the reduced intrasubject consistency during crawling could indicate that motor dysfunction in CDD group was the result of impaired motor control of the CNS in infant’s early life, and thus might provide an insight into the possible warning of neurological diseases, such as CP, in infant’s early life.

## Conclusion

In this study, kinematic synergy analysis was conducted in TD infants and infants with MDD during crawling. The present results reveal that, interlimb coordination patterns during infant crawling are the combinations of four types of single-limb movements, which represent the dominance of each of the four limbs (i.e., LU-, RU-, LL- and RL-dominant synergies). MDD mainly reduces the stability of joints and limbs, and induces the abnormal activation levels of limb-dominant synergies. In addition, MDD generally reduces the intrasubject consistency, especially in CDD group. Our preliminary work suggested that, these features based on kinematic synergies during infant crawling are promising to evaluate abnormal interlimb coordination in assisting the clinical diagnosis and motor rehabilitation of MDD. The main limitation of this study is that it is restricted to joint activities, which may be insufficient to accurately assess motor function of infants if muscular activities are not considered alongside they. In future work, we will further investigate these features in the joint and muscular activities during infant crawling, and recruit more infants with different motor developmental levels but similar biological ages to verify the effects of MDD on interlimb coordination.

## Methods

### Participants

Our experiments were conducted in collaboration with the Department of Rehabilitation Center, Children's Hospital of Chongqing Medical University, and recruited 40 infants with MDD (Table 2). The inclusion criteria for infants with MDD included: (1) biological ages of no more than 36 months; (2) prematurity, low birth weight, neonatal seizures, or other risk factors that might lead to motor dysfunction; (3) crawling continuously on their hands and knees during the experiments. Infants with MDD were excluded if they met any of following criteria: (1) malnutrition, genetic diseases, or other factors aside from brain injury that might affect motor function; (2) hands-and-feet crawling, step-crawl mix, creeping, or other atypical crawling postures; (3) halting, discontinuous hands-and-knees crawling. In addition, 20 TD infants were recruited from the hospital as the “control group” (Table 2). The inclusion criteria for TD infants included: (1) biological ages ranging from 8 to 15 months; (2) full-term birth with normal birth weight and no other neurological diseases affecting motor function; (3) crawling continuously on their hands and knees during the experiments. The exclusion criteria for TD infants were the same as exclusion criteria (2) and (3) for infants with MDD.

**Table 2:** Participant demographic information

Group	Sex	Biological age (months)	Delayed age of gross motor^a^ (months)	Diagnosis	Number of valid trails	Number of valid crawling cycles
TD (*N* = 20)	15 M, 5F	11 (3)	0 (2)	Normal (20)	3 (1)	5.5 (2.25)
ARDD (*N* = 16)	13 M, 3 F	11 (2)	1 (1)	MDD (15), MDD + EPI (1)	2 (0)	5 (2.25)
AHRDD (*N* = 11)	7 M, 4 F	16 (3)	5 (1)	MDD (6), CP (4), MDD + EPI (1)	2 (1.5)	5 (4)
CDD (*N* = 13)	6 M, 7 F	20 (9)	10 (4)	MDD (5), GDD (1), CP (6), CP + EPI (1)	3 (1)	8 (4)

^a^Denotes the value calculated from GMFM-88. Data are expressed as median (interquartile range). M: male, F: female, EPI: epilepsy. The diagnosis part is shown as disease (number of infants)

This study was reviewed and approved by the Ethics Committee of Children’s Hospital of Chongqing Medical University (approval code: 065/2011), and written informed consent was obtained from every infant's parent or guardian, in accordance with the Declaration of Helsinki.

### Clinical assessment

For every infant participating in our experiments, the developmental levels of motor function were assessed by the therapists from the Department of Rehabilitation Center, Children’s Hospital of Chongqing Medical University via GMFM-88. GMFM-88 measures the changes over time in gross motor function, including five dimensions: lying/rolling, crawling/kneeling, sitting, standing and walking/running/jumping [12]. Every dimension is scaled in a percentage score with range of 0–100. Meanwhile, GMFM-88 assesses the developmental age of gross motor for infants/children.

Based on GMFM-88, the pediatricians from the Department of Rehabilitation Center, Children’s Hospital of Chongqing Medical University recommended to identify the motor developmental levels of all infants by calculating the delayed ages (in months) of gross motor (= biological ages–developmental age of gross motor), and accordingly, further divided all 40 infants with MDD into 3 subgroups: ARDD (16 infants with a delayed age of gross motor of ≤ 3 months), AHRDD (11 infants with a delayed age of gross motor of 3–6 months) and CDD (13 infants with a delayed age of gross motor of > 6 months) groups (Table 2). Detailed contents about the grouping of those infants with MDD has been previously published in [33].

### Experimental protocol

**Fig. 5:**
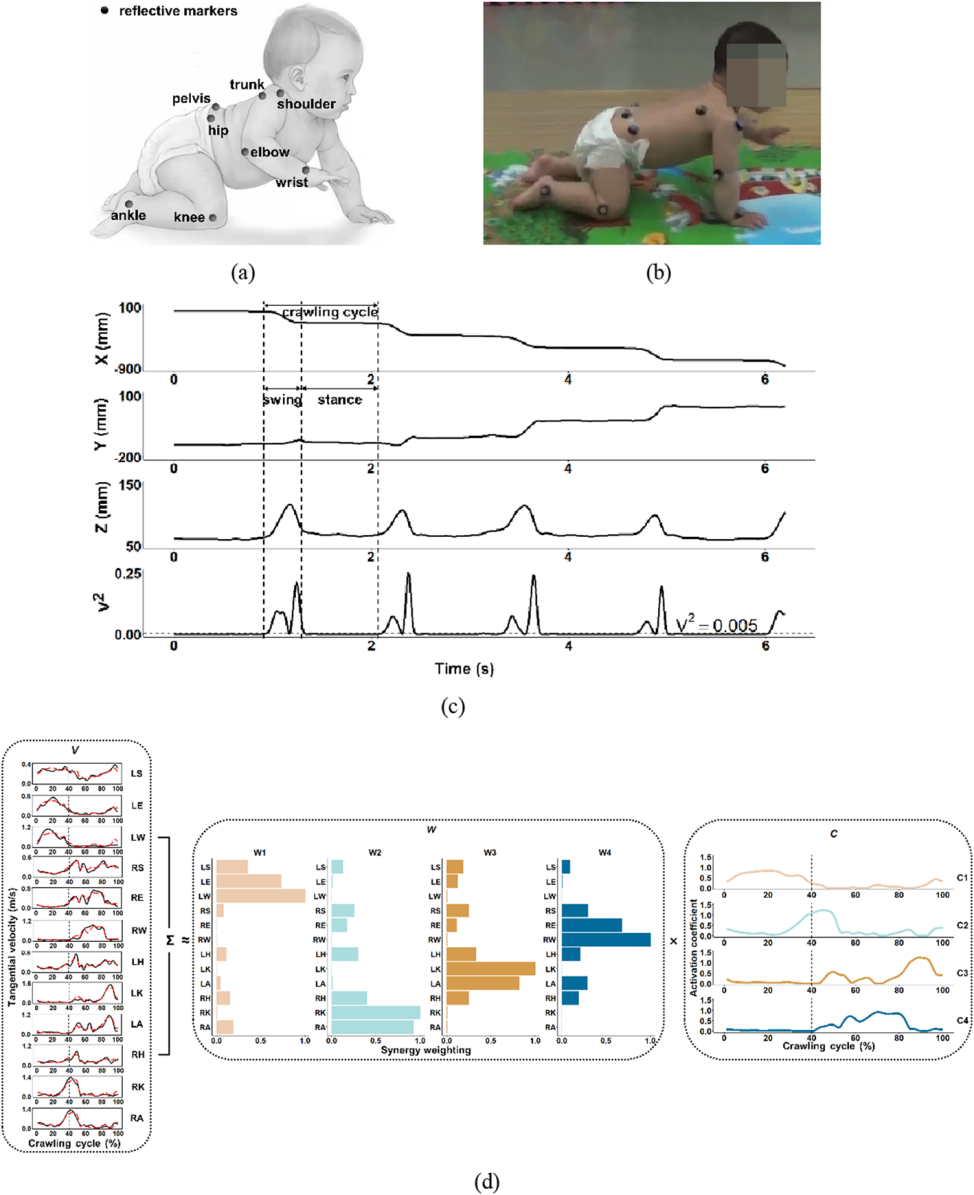
**a** Placement of reflective markers. **b** Experimental snapshot, (**c**) trajectories of the left wrist in *X*–*Y*–*Z* coordinates and the squared time derivative (${\text{V}}^{2}$, unit of velocity: m/s) of *Z* coordinates, as well as (**d**) a schematic illustration how tangential velocities of joints (${\text{V}}$) are decomposed into kinematic synergies (${\text{W}}$) and corresponding activation coefficients (${\text{C}}$) for a TD infant during crawling. Vertical dotted lines separate the swing and stance phases during a crawling cycle. For ${\text{V}}$ of (*d*), the black solid lines represent the original tangential velocities of joints, and the red dotted lines represent the reconstructed tangential velocities of joints resulting from the four kinematic synergies (i.e., W1, W2, W3 and W4) and corresponding activation coefficients (C1, C2, C3 and C4)

During infant crawling, kinematic data were recorded at 100 frames/s by a motion capture system (Raptor-E, Motion Analysis Corporation, USA) with 6 high-speed digital cameras in the three-dimensional (3D) gait analysis room, Department of Rehabilitation Center, Children’s Hospital of Chongqing Medical University. Twelve reflective markers were attached to the bilateral shoulder (lateral to the acromion), elbow (lateral epicondyle), wrist (ulnar styloid process), hip (posterior superior iliac spine), knee (lateral condyle of femur) and ankle (lateral fibular malleolus), and two reflective markers were taped over the pelvis (midpoint of bilateral posterior superior iliac spine) and trunk (right scapula), respectively (Fig. 5a).

Before the experiments, infants were required to spend time crawling on a mat (size 360 cm × 120 cm) placed on the floor to warm up and adapt to the experimental environment. Then, they were only allowed to wear underwear or diapers to minimize the obscuration of joints. During the experiments, they were encouraged to crawl at self-selected velocities from one end of the mat to the other (Fig. 5b). In this process, a set of personalized 3D trajectories of joints and limbs was precisely established for every infant. Detailed information about the experimental procedure has been published previously [33].

In this study, although several trials for every infant were recorded, only trials containing the kinematic data of all joints were taken into account. A valid crawling trial contained at least three complete and consecutive cycles, and the number of valid trails for the four groups were listed in Table 2.

### Data analysis

To investigate interlimb coordination during infant crawling, tangential velocities of joints were computed from recorded 3D joint trajectories, and kinematic synergies and corresponding activation coefficients were extracted from those joint velocities using the NMF algorithm. Then, the calculated VAF values and NMF-derived synergy weightings of each joint were used to measure the stability of joints and limbs. Moreover, the FWHM and CoA values of activation coefficients were calculated to quantify the activation levels of those synergies, and the CS values of those synergies and activation coefficients were employed to measure the intrasubject consistency.

#### Preprocessing

Kinematic data were first processed to interpolate for missing data over small time intervals using cubic spline interpolation. Locally weighted scatterplot smoothing was simultaneously used to remove noise. Then, they were low-pass filtered using a zero-lag fourth-order Butterworth filter with a cutoff frequency of 6 Hz to remove high frequency noise.

Before further analysis, the crawling cycles were segmented from the interpolated, smoothed and filtered kinematic data. A crawling cycle was defined as the time interval between two consecutive instances of lifting the same limb off the ground [18, 19]. In this study, we selected the left wrist as the detected object to segment the crawling cycle. In other words, the crawling cycle, including the swing and stance phases, was determined by computing the squared time derivative (i.e., square of velocity, unit of velocity: m/s) of Z coordinates of the left wrist, and each crawling cycle began with the swing phase [11, 14]. To identify the swing and stance phases, a threshold of 0.005 was selected based on our preliminary experiments (Fig. 5c). For every infant, the number of valid crawling cycles varied from 3 to 15 (Table 2), depending on his/her motor ability and the degree of participation.

#### Transformation from coordinates to tangential velocities

During hands-and-knees crawling, the joints of limbs perform spatial motions, so the motion of each joint can be decomposed into three mutually perpendicular velocities in the 3D space [6]. In addition, the tangential velocities of each joint are composed of those three velocities [27]. In this study, the tangential velocity of the ${\text{j}}$th joint at the ${\text{i}}$th time (${\text{V}}_{\text{i}}^{\text{ j}}$) is defined by the following equations:

1 $$V_{{x_{i} }}^{j} = \frac{{x_{i + 1} - x_{i - 1} }}{2\Delta t}$$

2 $${\text{ V}}_{{y_{i} }}^{{\text{ j}}} { = }\frac{{y_{{i{ + 1}}} - y_{i - 1} }}{{{2}\Delta t}}$$

3 $${ }V_{{z_{i} }}^{ j} { = }\frac{{z_{i + 1} - z_{i - 1} }}{{{2}\Delta t}}$$

4 $${\text{V}}_{\text{i}}^{\text{ j}}\text{ = }\sqrt{{\left({\text{V}}_{{\text{x}}_{\text{i}}}^{\text{ j}}\right)}^{2}\text{ + }{\left({\text{V}}_{{\text{y}}_{\text{i}}}^{\text{ j}}\right)}^{2}\text{ + }{\left({\text{V}}_{{\text{z}}_{\text{i}}}^{\text{ j}}\right)}^{2}}$$

where ${\text{x}}_{\text{i}}$, ${\text{y}}_{\text{i}}$ and ${\text{z}}_{\text{i}}$ are the 3D coordinates of any joint, ${\text{V}}_{{\text{x}}_{\text{i}}}^{\text{ j}}$, ${\text{V}}_{{\text{y}}_{\text{i}}}^{\text{ j}}$ and ${\text{V}}_{{\text{z}}_{\text{i}}}^{\text{ j}}$ are three joint velocities in the 3D space, and $\Delta t$ is the time interval between the ${\text{i}}$th and (${\text{i}}+ \text{1}$)th coordinates. Thereby, for each infant, the tangential velocities of the *j*th joint during a crawling cycle (${\text{V}}^{\text{ j}}$) were composed of a $1\; \times \;N$ sub-matrix, where ${\text{N}}$ is the length of a crawling cycle.

5 $$V^{ j} = \left[ {\begin{array}{*{20}c} {V_{1}^{{\text{ j}}} } & \cdots & {V_{N}^{{\text{ j}}} } \\ \end{array} } \right]$$

#### Kinematic synergy derivation

To allow the performance comparison across different crawling cycles, the tangential velocities of every joint during a crawling cycle (${\text{V}}^{\text{ j}}$) were resampled from 0% to 100% (increment: 1%), and the swing and stance phases were set to 40% and 60%, respectively [18]. Then, for each infant, all 12 submatrices (${\text{V}}^{\text{ j}}$) were concatenated into a 12 × (1 × 100) matrix (${\text{V}}$) arranged in the order shown in Fig. 5d to represent the tangential velocities of joints during a crawling cycle:

6 $$V{ = }\left[ {\begin{array}{*{20}c} {V^{{ 1}} } \\ \vdots \\ {V^{{\text{ j}}} } \\ \vdots \\ {V^{{ 12}} } \\ \end{array} } \right]{ = }\left[ {\begin{array}{*{20}c} {V_{1}^{{ 1}} } & \cdots & {V_{i}^{{ 1}} } & \cdots & {V_{{{100}}}^{{ 1}} } \\ \vdots & \ddots & \vdots & \ddots & \vdots \\ {V_{1}^{ j} } & \cdots & {V_{i}^{{\text{ j}}} } & \cdots & {V_{{{100}}}^{ j} } \\ \vdots & \ddots & \vdots & \ddots & \vdots \\ {V_{1}^{{ 12}} } & \cdots & {V_{i}^{{ 12}} } & \cdots & {V_{{{100}}}^{{ 12}} } \\ \end{array} } \right]$$

where the subscript (i.e., column variable) and superscript (i.e., row variable) represent the number of timepoints and joints, respectively. Then, NMF algorithm was performed on the tangential velocity matrix (${\text{V}}$) to derive kinematic synergies. Briefly, this algorithm used an iterative method that minimized the sum of squared error (SSE) between the original matrix (${\text{V}}$) and the reconstructed matrix ($\widetilde{\text{V}}$) to approximately decompose each matrix (${\text{V}}$) into two non-negative matrices: the kinematic synergy matrix (${\text{W}}$) and the activation coefficient matrix (${\text{C}}$) [17, 23] (Fig. 5d):

7 $${ }V^{{ {12}\; \times \;{100}}} = \tilde{V}^{{{ 12}\; \times \;{100}}} { + }\varepsilon$$

8 $$\tilde{V}^{{{ 12}\; \times \;{100}}} { = }W^{{{ 12}\; \times \;m}} C^{{ m\; \times \;{100}}}$$

where ${\text{m}}$ is the specified number of kinematic synergies ($1 \le m \le {12}$), and $\varepsilon$ is the residual between ${\text{V}}$ and $\widetilde{\text{V}}$. Each column of ${\text{W}}$ represents the relative weightings across all joints in each synergy, and each row of ${\text{C}}$ represents the activation pattern of corresponding synergy during a crawling cycle. Because the SSE converged to a local minimum, the SSE was repeatedly calculated until the maximum number of iterations reached 1000 or the value of SSE was fell below ${\text{e}}^{ \, -{6}}$.

To determine the minimum number of kinematic synergies that best accounted for the variance of the data, the VAF values (ranging from 0 to 100%) were calculated as follows.

9 $${\text{VAF = }}\left( {{1}\; - \;\frac{{\varepsilon^{2} }}{{V^{2} }}} \right)\; \times \;{\text{100\% }}$$

In this study, the best number of kinematic synergies for each infant satisfied the following criteria: (1) the overall reconstructed tangential velocities accounted for more than 90% of the variance for all joints (i.e., total VAF > 90%); (2) the reconstructed tangential velocities of each joint accounted for more than 75% of the variance for the corresponding joint (i.e., joint VAF > 75%); (3) the increase in mean values of joint VAF for all joints was less than 5% (i.e., $\Delta \left( {\frac{1}{{{12}}}\mathop {\sum \;}\nolimits {\text{joint}}\;{\text{ VAF}}} \right){\text{ < 5\% }}$. With these criteria, this algorithm was conservative enough to ensure good reconstruction of the data [9, 45]. At the same time, larger joint VAF values and synergy weightings indicted higher stability of joints and limbs during infant crawling [8, 27].

#### Activation levels of kinematic synergies

To quantitatively analyze the activation duration and activation timing of kinematic synergies, the FWHM and CoA values of activation coefficients were calculated separately. In this study, for the activation coefficients of every order kinematic synergy of tangential velocities of joints during a crawling cycle, the FWHM value was calculated as the time interval (in % of a crawling cycle) in which those coefficients (after their minimum was subtracted) exceeded half of their maximum [9, 25], using the following equations:

10 $${\text{BA }} = v\left( {{\text{if}}\;c_{v} > \frac{{\max {-} \min }}{2} \;{\text{and}}\;c_{v - 1} < \frac{{\max {-} \min }}{2}} \right)$$

11 $${\text{EA}} = u\left( {{\text{if}}\;c_{u} > \frac{{\max {-} \min }}{2}\;{\text{ and}}\;c_{u - 1} < \frac{{\max {-} \min }}{2}} \right)$$

12 $$\text{FWHW = EA }-\text{ BA + 1}$$

where BA and EA are the beginning and ending moments of the kinematic synergy being activated, respectively. In addition, ${\text{max}}$ and ${\text{min}}$ are the maximum and minimum of activation coefficients, respectively; ${\text{c}}_{\text{u}}$ and ${\text{c}}_{\text{v}}$ are the ${\text{u}}$th and ${\text{v}}$th values of activation coefficients, respectively.

For the activation coefficients of every order kinematic synergy of tangential velocities of joints during a crawling cycle, the CoA value was calculated using the circular statistics as the angle of these coefficients in polar coordinates (denoting the phases during a crawling cycle, with angle $1 \le \theta_{i} \le 2\pi$) that pointed to the center of mass of this circular distribution [9, 30, 31], as follows:

13 $${\text{A = }}\mathop \sum \limits_{{i{ = 1}}}^{t} \left( {{\text{cos}}\theta_{i} \times c_{i} } \right)$$

14 $$B{ = }\mathop \sum \limits_{{i{ = 1}}}^{t} \left( {{\text{sin}}\theta_{i} \times c_{i} } \right)$$

15 $$\text{CoA = arctan}\left(\text{B/A}\right)$$

where ${\text{t}}$ (${\text{t}}\text{ = 100}$) is the number of timepoints during a crawling cycle, and ${\text{c}}_{\text{i}}$ is the ${\text{i}}$th value of activation coefficients. Larger FWHM and smaller CoA values indicated greater immaturity of crawling movement [9].

#### Intrasubject consistency

For periodic human motor behaviors, the CS can quantify the intrasubject consistency [32]. By importing the CS to kinematic synergy analysis, the intrasubject consistency can be evaluated by separately calculating the similarity of previously sorted kinematic synergies and activation coefficients [10]. CS values range from 0 (no similarity) to 1 (complete similarity), and larger CS values indicate higher intrasubject consistency [10, 32]. In this study, for every infant, the CS values of kinematic synergies (${\text{CS}}_{{\text{W}}_{\text{m, n}}^{\text{ k}}}$) were calculated by any two ${\text{k}}$-order synergy weighting vectors in the ${\text{m}}$th and ${\text{n}}$th kinematic synergy matrices (${\text{W}}_{\text{m}}^{\text{ k}}$ and ${\text{W}}_{\text{n}}^{\text{ k}}$), and the CS values of activation coefficients ($CS_{{C_{m,n}^{k} }}$) were the normalized scalar products of corresponding ${\text{k}}$-order activation coefficient vectors (${\text{C}}_{\text{m}}^{\text{ k}}$ and ${\text{C}}_{\text{n}}^{\text{ k}}$).

16 $${\text{CS}}_{{\text{W}}_{\text{m, n}}^{\text{ k}}}\text{ = }\frac{{\text{W}}_{\text{m}}^{\text{ k}} \cdot {\text{W}}_{\text{n}}^{\text{ k}}}{\Vert {\text{W}}_{\text{m}}^{\text{ k}}\Vert \cdot \Vert {\text{W}}_{\text{n}}^{\text{ k}}\Vert }$$

17 $${\text{CS}}_{{\text{C}}_{\text{m, n}}^{\text{ k}}}\text{ = }\frac{{\text{C}}_{\text{m}}^{\text{ k}} \cdot {\text{C}}_{\text{n}}^{\text{ k}}}{\Vert {\text{C}}_{\text{m}}^{\text{ k}}\Vert \cdot \Vert {\text{C}}_{\text{n}}^{\text{ k}}\Vert }$$

### Statistical analysis

Descriptive statistics include mean and standard deviation for normal distributions, and median and interquartile range for non-normal distributions [1, 5]. In general, Shapiro–Wilk test is used to test the normality of the data in small samples (sample size ≤ 50), whereas Kolmogorov–Smirnov test is applied to test the normality of the data in relatively large samples (sample size > 50). Comparative statistics use the parametric tests if data sets are normally distributed and of equal variance; otherwise, nonparametric tests are used [1, 5]. In this study, Shapiro–Wilk test was used to test the normality of biological ages and delayed ages of gross motor for the four groups, whereas Kolmogorov–Smirnov test was applied to all other data sets. Paired *t* test and Wilcoxon signed-rank test were used for intragroup comparisons of joint VAF values of any two joints in each of the four limbs, synergy weightings of any two main activated joints in each order kinematic synergy, and FWHM, CoA, CS values of any two order kinematic synergies. Kruskal–Wallis one-way ANOVA on ranks (with a Tukey post hoc test) was used for intergroup comparisons of the best number of kinematic synergies, joint VAF values of each joint, synergy weightings of each main activated joint, and FWHM, CoA, CS values of each kinematic synergy. In the above tests, *p* < 0.05 was considered statistically significant. All statistical analyses were conducted using the IBM SPSS, version 24.0 (SPSS Inc., Chicago, IL, USA).

## Data Availability

The data sets generated and/or analyzed during the current study are not publicly available due to clinical policy, but are available from the corresponding author upon reasonable request.
